# A Comprehensive Review on Chickpea (*Cicer arietinum* L.) Breeding for Abiotic Stress Tolerance and Climate Change Resilience

**DOI:** 10.3390/ijms23126794

**Published:** 2022-06-18

**Authors:** Osvin Arriagada, Felipe Cacciuttolo, Ricardo A. Cabeza, Basilio Carrasco, Andrés R. Schwember

**Affiliations:** 1 Departamento de Ciencias Vegetales, Facultad de Agronomía e Ingeniería Forestal, Pontificia Universidad Católica de Chile, Santiago 7820436, Chile; arriagada.lagos.o@gmail.com (O.A.); facacciuttolo@uc.cl (F.C.); 2 Departamento de Producción Agrícola, Facultad de Ciencias Agrarias, Universidad de Talca, Talca 3460000, Chile; rcabeza@utalca.cl; 3 Centro de Estudios en Alimentos Procesados (CEAP), Av. Lircay s/n, Talca 3480094, Chile; bcarrasco@ceap.cl

**Keywords:** chickpea, breeding, QTL, abiotic stress, omics

## Abstract

Chickpea is one of the most important pulse crops worldwide, being an excellent source of protein. It is grown under rain-fed conditions averaging yields of 1 t/ha, far from its potential of 6 t/ha under optimum conditions. The combined effects of heat, cold, drought, and salinity affect species productivity. In this regard, several physiological, biochemical, and molecular mechanisms are reviewed to confer tolerance to abiotic stress. A large collection of nearly 100,000 chickpea accessions is the basis of breeding programs, and important advances have been achieved through conventional breeding, such as germplasm introduction, gene/allele introgression, and mutagenesis. In parallel, advances in molecular biology and high-throughput sequencing have allowed the development of specific molecular markers for the genus *Cicer*, facilitating marker-assisted selection for yield components and abiotic tolerance. Further, transcriptomics, proteomics, and metabolomics have permitted the identification of specific genes, proteins, and metabolites associated with tolerance to abiotic stress of chickpea. Furthermore, some promising results have been obtained in studies with transgenic plants and with the use of gene editing to obtain drought-tolerant chickpea. Finally, we propose some future lines of research that may be useful to obtain chickpea genotypes tolerant to abiotic stress in a scenario of climate change.

## 1. Introduction

Chickpea (*Cicer arietinum* L.) is the third most important pulse crop worldwide, with a cultivated area of 14.84 million hectares, a production of 15.08 million tons, and an average yield of 1.01 t/ha in 2020 [1], which is significantly lower than the estimated potential of 6 t/ha under optimum growing conditions [2]. Chickpea is cultivated mainly in arid and semi-arid areas in more than 50 countries across the Mediterranean basin, Central Asia, East Africa, Europe, Australia, and North and South America [3], where it is highly vulnerable to abiotic stresses such as drought and heat at various growth stages during the productive season [4]. Chickpea is mainly produced in developing countries, where more than 90% of chickpea production is consumed locally [5]. The main chickpea-producing and consuming region is the Indian subcontinent (India, Pakistan, Myanmar, Bangladesh, and Nepal), contributing almost 70% of the world’s production [5]. In addition, Turkey, Australia, Ethiopia, Iran, Mexico, Canada, and the USA are other countries with high chickpea production [6].

Globally, more than 2.3 billion people, or 30% of the worldwide population, are affected by one or more forms of malnutrition [1,7], which is strongly related to inadequate food intake and low nutrient content of foods [8]. Chickpea is a good source of protein (higher than cereal grains), dietary fiber, beneficial unsaturated fatty acids, vitamins, and macro and micro-nutrients, and has several health benefits for an expanding world population [9]. In this context, this legume plays a key nutritional role in the diet of millions of people in the world, helping to fight several health issues, such as cardiovascular disease, type 2 diabetes, digestive diseases, and some cancers [6,10].

On the other hand, chickpea is cultivated mainly in arid and semi-arid environments on soils of poor agricultural quality [11]. In these regions, the abiotic stresses, such as extreme temperatures and drought at various growth stages during the productive season, are the major adverse environmental factors that limit its production [12]. In fact, drought and extreme temperatures account for up to 50% and 20%, respectively, of chickpea yield losses in the world [13]. Under this scenario, the identification and/or development of highly productive chickpea genotypes by breeders—through a combination of breeding approaches—is critically necessary. These new chickpea cultivars must be climate change resilient, genetically diverse, efficient, and widely adaptable to a range of environments to maintain food security in the near and medium-term future [14]. In this sense, the main objective of this work is to provide a comprehensive review of conventional and modern breeding approaches currently applied to develop chickpea cultivars tolerant to different abiotic factors, mainly drought, salinity, and extreme temperatures.

## 2. Origin, Domestication, and Germplasm Diversity in Chickpea

**Figure 1:**
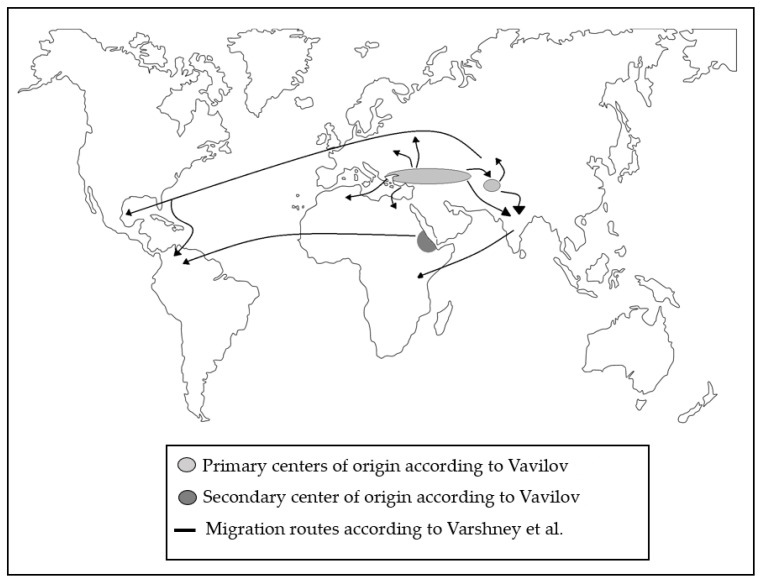
Zones of origin of cultivated chickpea (*C. arietinum*) and subsequent distribution around the world. Primary centers of origin according to Vavilov [17], Secondary center of origin according to Vavilov [17], Migration routes according to Varshney et al. [19]. Adapted from Croser et al. [20].

Chickpea belongs to the genus *Cicer*, which comprises 10 annual and 36 perennial species; among the annual species, *C. arietinum* is the only domesticated and cultivated species worldwide [15]. Archaeological remains of chickpea were found in the Middle East back to 7500–6800 BC [16]. In this context, Vavilov suggested, as primary centers of chickpea origin, the Fertile Crescent (presently Southeastern Turkey and Syria; Figure 1) and the Mediterranean region, whereas the secondary centers of origin are South Asia and Ethiopia [17,18]. Recently, a comprehensive study based on whole-genome resequencing of 429 lines sampled from 45 countries suggests that the chickpea migration route occurred from the Mediterranean region/Fertile Crescent to South Asia (India) and then to East Africa and Central Asia in parallel. In addition, the migration to America occurred from Central Asia or Eastern Africa rather than the Mediterranean basin alone [19].

**Figure 2:**
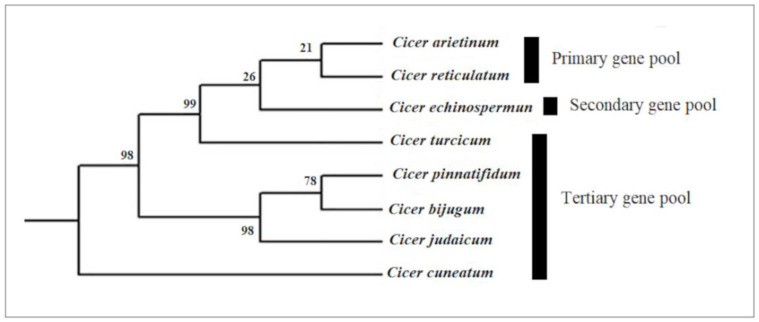
Phylogenetic tree from the maximum parsimony analysis based on ITS region in annual *Cicer* species belonging to different gene pools, adapted from Toker et al. [15].

Currently, wild annual *Cicer* species are grouped into three gene pools (primary, secondary, and tertiary) based on the studies considering the crossability and fertility of hybrids in interspecific crosses with cultivated chickpeas, reflecting their genetic distance from the cultivated species [20]. The unsynchronized growth of the stigma and anthers is one of the main interspecific hybridization barriers. Limited compatibility leads to impaired meiosis and imbalanced gamete formation and, therefore, the production of infertile hybrids [15,19]. The primary gene pool includes the cultivated chickpea *C. arietinum* and its immediate progenitor species, *C. reticulatum*, which is readily crossable with *C. arietinum;* their progeny is fully fertile due to good chromosomal pairing [15]. Therefore, it serves as a potential source to broaden the genetic base and enhance the yield components and stress tolerance traits in the cultivated gene pool [21,22,23]. The secondary gene pool comprises *C. echinospermum,* which is crossable with the cultivated species, but with reduced fertility of the resulting hybrids and subsequent progenies. Consequently, these species are potential donors of valuable alleles/genes for chickpea improvement. The tertiary gene pool consists of the remaining Cicer species, including annual species such as *C. bijugum*, *C. judaicum*, *C. pinnatifidum*, and *C. cuneatum*. These species cannot be easily crossed with cultivated chickpeas through conventional breeding methods [24]. The phylogenetic relationship between the annual Cicer species belonging to the three different gene pools is shown in Figure 2, which was extracted from a recent study based on internal transcribed spacer (ITS) sequences in *Cicer* taxa [15].

To understand the molecular diversity among wild and cultivated Cicer species, the process of genetic improvement can be sped up through interspecific hybridization [23]. In this sense, many studies have been performed to reveal genetic diversity patterns, population structure, and phylogenetic relationships among diverse annual and perennial *Cicer* species using different types of molecular markers such as AFLP [22,25], SSR [26,27], and SNP [22,24,28]. Higher levels of genetic variation have been observed across wild Cicer species in those studies. The highest levels of genetic diversity were reported in populations from South Asia, while the lowest levels were found in populations from America, which is in accordance with the process of origin and migration of the chickpea [24]. In addition, populations from the Fertile Crescent had greater genetic diversity than those from Central Asia [22]. However, the genetic diversity of cultivated chickpea is very narrow because of several bottlenecks occurring during its domestication process [29]. The average yield of chickpea is relatively low, with a mean grain yield of 1.01 t/ha in 2020 [1], which indicates the need to genetically improve the yields and achieve the desired productive goals by growers [30]. Knowledge of the level of genetic variation within and among populations of chickpea is essential for any breeding program. In fact, the natural genetic variability is key to responding to the effects of climate change on crop yields [31]. Given the series of ‘genetic bottleneck’ events that occurred during the domestication process, the major constraint in any chickpea breeding program is its narrow genetic base, which makes it difficult for breeders to produce new elite chickpea cultivars with long-lasting tolerance to major abiotic stresses [32]. Therefore, to make breeding efforts more effective, a broadening of the genetic base of chickpea is very required [30].

The world collection of chickpea germplasm comprises 99,877 accessions, including 1476 wild Cicer types, which are preserved in 120 genebanks spread across 64 nations [30]. The three main chickpea germplasm banks are the International Crops Research Institute for the Semi-Arid Tropic (ICRISAT) with 18,963 accessions in India, the National Bureau of Plant Genetic Resources (NBPGR) in India with 15,986 accessions, and the International Center for Agricultural Research in the Dry Areas (ICARDA) in Lebanon with 13,065 accessions [33]. This large number of accessions represents a great source of favorable alleles that can be incorporated into breeding programs related to grain yield and abiotic stress tolerance [30]. In chickpea, hundreds of high-yielding cultivars tolerant to major abiotic stresses have been developed through conventional breeding methods [34]. A list of the chickpea genotypes tolerant to drought, heat, cold, salt, and stress is reported in the previous works carried out by Jha et al. [35] and Rani et al. [36].

## 3. Impact of Abiotic Stresses in Chickpea

The global chickpea production is mainly limited by biotic and abiotic stresses. Among the abiotic factors, drought at the terminal stage is the most important constraint to yield in chickpea, accounting for about 50% of yield losses globally [37]. It is known that climate change has a negative impact on crop production; in fact, the chickpea yield decreased by 38.5 kg/ha with a rise in temperature by 0.1 °C combined with a 31% reduction in seasonal rainfall [38]. All of the essential biological processes, including photosynthesis, respiration, transpiration, and other essential biochemical processes, are seriously affected by drought stress [35]. Four different mechanisms of response to drought stress in plants have been identified, which can be divided into avoidance, tolerance, escape, and recovery. Early phenology is the most important mechanism for escaping terminal drought stress. In addition, drought avoidance can be achieved by water uptake by the roots from deeper soil layers, osmotic adjustment, and reducing water loss (stomata conductance or leaf area reduction). For more details on these mechanisms of response to drought stress in chickpea, please see Fang and Xiong [39] and Maqbool et al. [40]. Different drought-related traits, such as root architecture, phenology traits, leaf traits, osmotic adjustment capabilities, water potential, ABA content, and stability of the cell membrane, have been used as indicators to evaluate the drought tolerance in plants [39]. In this sense, the development of cultivars that can escape, avoid, or tolerate drought should be the main objective of chickpea breeding programs.

The average temperature on Earth is approximately 14 °C, and according to the Intergovernmental Panel on Climate Change, the average global temperature will rise by 1.8 to 4.0 degrees over the next century [41]. This increase in temperature causes a shift in seasons that significantly affect chickpea production. For example, the optimal temperatures for chickpea growth range between 10 °C and 30 °C [42], and yield loss was estimated to be between 10% and 15% for every 1 °C above the optimum growth temperature [43]. Exposure to heat stress (>35 °C) during reproductive development drastically reduced the yields, resulting in yield losses of up to 39% due to male reproductive tissue (pollen and anther) functions being negatively affected [44]. Heat stress affects different growth stages ranging from germination to grain yield [12]. Moreover, a variety of physiological processes such as photosynthesis, respiration, transpiration, membrane thermostability, and osmotic regulation are adversely affected by heat stress [45]. One of the major consequences of heat stress is the excess generation of reactive oxygen species (ROS), which leads to oxidative stress [46]. Different mechanisms have been identified to minimize heat stress damage during flowering in plants. These include heat escape, where flower initiation and plant maturation occur early and rapidly so that plants mature before the onset of high temperatures; heat avoidance, through transpiration cooling, leaf reflectance, and orientation to avoid the direct impacts of the sun; and heat tolerance, through resilient reproductive processes [47]. Regarding this last mechanism, the production of compatible solutes that can organize proteins and cellular structures, maintain cell turgor by osmotic adjustment, and modify the antioxidant system to re-establish the cellular redox balance and homeostasis are some of the response mechanisms to heat stress in plants [46]. Recently, the work conducted by Jeffrey et al. [48] addresses in detail the response mechanisms of chickpea to heat stress, emphasizing that most studies are focused on improving heat tolerance through earliness to avoid stress. However, with rapidly changing environmental conditions, more effort is required to achieve true heat tolerance rather than avoidance.

In contrast, chickpea also suffers from cold stress when it faces chilling (3–8 °C) or even freezing temperatures that result in arresting the germination process and affecting seedling vigor negatively during early growth establishment [35]. Phenotypic effects, such as poor germination, stunted seedlings, yellowing of leaves (chlorosis), reduced leaf expansion and wilting, and death of tissue (necrosis), are symptoms of plant exposure to cold stress [49]. In the reproductive stage, flower abortion and inhibition of pollen tube growth are also cold stress effects [50]. However, the main negative effect of cold stress on the plant is that it causes severe membrane damage due to cellular dehydration associated with freezing during cold stress [49]. Therefore, preventing the formation of intracellular ice crystals is of great importance for plants to tolerate cold stress.

Soil salinity stress is one of the growing problems worldwide due to improper agricultural land and irrigation practices resulting in high concentrations of toxic ions (Na^+^ and Cl^−^) in arable land [51]. The excess of soluble salts in the soils leads to osmotic stress and ion toxicity that results in ionic imbalance and can cause plant death [52]. Chickpea is severely affected by soil salinity, which results in significant yield losses. Some of the effects of salinity on biological processes are damage to photosystem II and nutritional imbalance, which leads to the reduction in germination, plant growth (biomass), and seed size [53]. Numerous adaptations such as osmoregulation and osmotic adjustment, hormonal regulation, activation of the antioxidant defense system, and ion homeostasis are some of the mechanisms involved in conferring salt tolerance (for more details, see Farooq et al. [54]). These mechanisms can be categorized as “ion exclusion”, to eliminate Na^+^ and Cl^−^ ions from roots when their accumulation becomes toxic; “tissue tolerance”, allowing the compartmentalization of toxic ions at the cellular and intracellular levels; and “osmotic tolerance” [35].

## 4. Breeding Approaches in Chickpea

The basis for applying any genetic improvement strategy is the use of the genetic diversity present in the species for the target traits and the development of improvement methods using Mendelian and quantitative genetic principles for efficient selection [55]. Breeding methods based on recombination (hybridization), mutation, and transgenic methods, among others, aim to create new genetic variability in the crops and then introduce the genes/alleles associated with the traits of interest into the new cultivars [56]. Therefore, the creation of genetic variability, phenotypic selection of desired individuals, and subsequent evaluation of selected lines are the three basic steps of a breeding program [30]. When the chickpea started to be genetically improved, the selection of native or introduced landraces was used by breeders to develop new chickpea cultivars, while the most recent cultivars were developed through hybridization [37]. However, these advances, using conventional breeding approaches, are still not satisfactory to ensure food security for the growing human population. The adoption of modern technologies and different breeding strategies can play an important role in increasing the rate of genetic gain for traits related to grain yield under stressful environmental conditions [4]. In this context, the development of genetic and genomic resources for chickpeas, such as the availability of many molecular markers, dense genetic maps, and the identification of QTLs, coupled with ‘omics’ technologies, have made it possible to improve grain yields and the adaptation of chickpea to abiotic stress [37,57]. Thus, the integration between conventional and modern breeding techniques is essential to accelerate the genetic gains of chickpea breeding programs.

### 4.1. Conventional Breeding in Chickpea

Knowledge of the genetic architecture of the traits of interest, such as the magnitude of heritability, genetic interactions, and genetic correlations between traits, is required for developing a successful breeding program [58]. In chickpea, moderate to low heritability has been reported for most yield-related traits such as grain yield, number of pods per plant, number of seeds per pod, and plant height [59,60]. Furthermore, moderate to low heritability has been estimated for traits related to abiotic stress tolerance, such as root system architecture, stomatal conductance, and canopy temperature, among others [61,62,63]. In fact, it is widely known that the traits related to yield components and abiotic tolerance are complex and involve numerous genes associated with their regulation. The phenotypic expression of the desired traits is then greatly affected by the genotype (G), the environment (E), and their interactions (G × E) [64]. Despite this, over the past 50 years, there has been a significant improvement in the yield and tolerance to abiotic stress in chickpea [36,65]. This progress has been achieved mainly using the conventional breeding approaches such as plant introduction, hybridization, and mutation, which allow the incorporation of genes/alleles associated with yield components and tolerance to abiotic stress into cultivars of chickpea [55]. The donor parent of these genes/alleles that confer some desired trait can be found among cultivars, germplasm collection, spontaneous or induced mutants, and/or wild relatives [66].

The introduction may involve new cultivars, landraces, wild relatives, or a new crop species for the area. This method makes it possible to increase genetic diversity and search for a desirable genotype with higher yield and better adaptability to the local environment [30]. In recent decades, great strides have been made in increasing the genetic diversity of cultivated chickpea through the sharing of germplasm across the world and the incorporation of crop wild relatives and landraces [23,67]. It is widely known that wild Cicer species such as *C. anatolicum* and *C. reticulatum* are an important reservoir of genes/alleles that are useful to increase productivity [20,68,69] and to generate tolerance to abiotic stresses such as drought and heat stress in the modern cultivars, as shown in Table 1 [69,70,71].

After the introduction of genotypes, hybridization between diverse and contrasting parents is performed to create variability in the progeny through the genetic recombination events [77]. It is important to note that the selection of appropriate parents is the key to success in any hybridization program through progeny tests [40]. Even though chickpea is a self-pollinating species, the rate of natural cross-pollination ranges from 10 to 50% [30]. Intraspecific and wide hybridization techniques have been mainly performed to improve the cultivated chickpea [33].

Several genes have been introgressed into cultivated chickpea through hybridization. For example, Singh and Ocampo [68] introgressed genes from *C. echinospermum* and *C. reticulatum* into *C. arietinum* and observed an up to 39% increase in grain yields. In addition, the introgression of genes from *C. reticulatum* to cultivated chickpea increased seed yield by 6.1–17% [20]. Regarding tolerance to abiotic stresses, a significant level of drought tolerance has been found in *C. pinnatifidum* and *C. reticulatum* [71,78]. In addition, Canci and Toker [71] showed that *C. reticulatum* and *C. pinnatifidum* resist heat stress up to 41.8 °C. In parallel, *C. bijugum*, *C. reticulatum*, *C. echinospermum,* and *C. pinnatifidum* have shown high tolerance to low temperatures [72,73,78,79]. However, there are no reports to date describing the introgression of genes associated with cold tolerance from wild to cultivated chickpea [23]. Due to genetic incompatibility, interspecific hybridization has restricted the use of only two wild Cicer species, *C. reticulatum* and *C. echinospermum*, which can readily be crossed with the cultivated chickpea *C. arietinum* [80]. In addition, *C. reticulatum* has been widely used as a donor parent due to its alleles showing disease resistance, drought, and heat stress [71], and having appropriate yield components [20]. More recent approaches to hybridization, such as the development of multiparent advanced generation intercross (MAGIC) populations, can be used to combine favorable alleles for tolerance to abiotic stresses and other desired traits [81]. In chickpea, a MAGIC population was developed at ICRISAT using eight improved cultivars and widely adaptable breeding lines. Several genotypes of the MAGIC population showed higher yield performance and tolerance to drought and heat stress than the best check; therefore, these genotypes provide a useful germplasm source with diverse allelic combinations for the improvement of global chickpea breeding programs [82].

Mutagenesis approaches are useful in situations in which the crops have a narrow genetic diversity, small flowers, and when they are very difficult to hybridize. Some of these developed mutants are released directly as cultivars, or they are used as donors to improve a specific trait [83]. Studies have used mutagenesis to improve tolerance to abiotic stresses. For example, ten accessions of *Cicer* species were irradiated with gamma rays to improve tolerance to drought, heat, and salinity stress, and most of the mutant lines were more tolerant to the concerned stresses than their parents [84]. In addition, Akhar et al. [85] used gamma rays to improve cold tolerance in two chickpea genotypes (MCC741 and MCC495). Mutagenesis has enabled diversification of the genetic variability of chickpea with the aim of increasing yield and tolerance to a wide range of stresses. According to the Joint FAO/IAEA Division of Nuclear Techniques in Food and Agriculture, a total of 27 mutant high-yielding cultivars of chickpea, some tolerant to abiotic stresses, have been developed to date and officially released (https://mvd.iaea.org/, accessed on 17 May 2022). Details of the chickpea cultivars developed and released through mutations are shown in Table 2.

### 4.2. Modern (Omics) Breeding Approach in Chickpea

Even though the conventional breeding methods have increased yields and tolerance to abiotic stresses, the main limitation of these procedures is that they are appropriate for highly heritable and easy-to-score/visualize traits [4]. The adaptive traits associated with tolerance to abiotic stresses and yield are multigenic, have low heritability, and are highly influenced by the environment [86]. Conventional breeding does not guarantee reaching the grain yield levels of chickpea required for the food security of the growing world population under increasingly extreme and stressful environments due to climate change [4]. Under this stage, modern (omics) breeding approaches can be used as a complement to increase selection efficiency, reduce breeding time, and identify the specific genes that control the desired traits in legumes [87]. Thus, integration between these breeding approaches is the key to developing high-yielding chickpea cultivars that are resilient to the consequences of climate change. Different types of omics, such as genomics, transcriptomics, proteomics, and metabolomics, have been used to characterize plant responses, at different molecular levels, to stressful environmental conditions.

#### 4.2.1. Genomics in Chickpea

Significant advances have been made in the development of genomic resources of chickpea during the past decade, which has allowed us to dissect the genetic architecture of several traits of interest related to tolerance and yield performance [87,88]. Genomics includes the development of molecular markers, which allow us to analyze genetic diversity, develop genetic maps, and identify regions of the genome (quantitative trait loci; QTL) associated with desirable traits in crops [89]. In the last decades, thousands of molecular markers have been developed in chickpea, including RAPD [90], SSR [91], DArT [92], and SNP [93] markers. Currently, the next-generation sequencing (NGS) and high-throughput genotyping technologies offer opportunities to generate large amounts of high-throughput data to capture millions of variations at the genome level. That is how a high-density ‘Axiom^®^*CicerSNP* Array’ with 50,590 nonredundant SNPs was developed [94]. The development of these markers has allowed the construction of several genetic maps, including high-density genetic maps in chickpea [95,96,97]. In addition, the availability of draft genome sequences [11,98], large-scale resequencing efforts [99], and with the detailed map of genetic variation of 3171 cultivated and 195 wild accessions of chickpea [100] have been important tools that have strengthened its genomics. The use and integration of these genomic resources will improve the precision and efficiency of breeding programs in chickpea. Many genomic regions or QTLs associated with tolerance to abiotic stresses (mainly drought, extreme temperatures, and salinity) and yield components have been documented through QTL and association mapping in chickpea. Table 3 summarizes some major QTL associated with tolerance to abiotic stresses identified in chickpea. Major QTL has been detected in all chickpea chromosomes, except on linkage group (LG) 2. Furthermore, important genomic regions on LG3 and LG4 have been associated with tolerance to abiotic stress, and LG4 has been extensively studied.

For tolerance to drought stress, Rehman et al. [61] identified 15 genomic regions significantly associated with traits affecting drought tolerance in an RIL population derived from the cross between ILC 588 (drought tolerant) and ILC 3279 (susceptible) genotypes of chickpea. A QTL on LG1 was associated with the grain yield and harvest index (HI), which explained up to 8% and 13% of the phenotypic variability for grain yield and HI, respectively. This region also influenced drought tolerance, explaining 12% of the total phenotypic variability. Another QTL on LG3 explained 25% and 27% of the phenotypic variability for HI and drought tolerance, respectively [61]. A comprehensive understanding of drought tolerance in chickpea was given by Varshney et al. [13]; they identified a “QTL-hotspot” region on GL4 of 29 cM containing 12 QTLs for 12 drought tolerance-related traits, which together explained 58.2% of the phenotypic variation. This QTL-hotspot has been introgressed into some new cultivars to increase drought tolerance and seed yield of chickpeas grown under environmental stress conditions [102,114,115]. Subsequently, using high-throughput genotyping technologies, this region on GL4 was refined to 14 cM equivalent to 3 Mb [104] and divided into two subregions, namely “QTL-hotspot_a” and “QTL-hotspot_b”, containing 15 and 11 candidate genes, respectively [105].

In the context of global climate change, the heat stress during the reproductive stages can severely affect the grain yields of chickpea [107]. In this sense, the identification and introgression of QTLs associated with heat tolerance can accelerate the breeding process and facilitate combining different desired traits in one single genotype. The genetic architecture of heat stress tolerance has been extensively studied in chickpea [106,116,117]. For example, Paul et al. [106] identified four major QTLs controlling pod and grain yield traits on LG5 and LG6 under heat stress, which explained above 50% of the phenotypic variation of those traits. Moreover, Jha et al. [117] reported a QTL associated with chlorophyll content on LG6 at ~100 cM, explaining 17.2% of the phenotypic variation under heat stress. A total of 37 major QTLs across the genome for 12 different traits related to heat tolerance were identified [117]. Finally, Kushwah et al. [118] identified 13 stable QTLs for 7 different traits, including days to flowering. Stable QTLs for days to flowering can be one of the major factors for providing heat tolerance since early flowering is an evasion mechanism that allows seeds to be produced earlier compared to plants with longer cycles [118], reflecting the convenience of developing early cultivars in chickpea breeding programs.

Conversely, limited genomic resources have been reported for cold tolerance in chickpea. According to Abbo et al. [29], wild chickpea accessions respond to vernalization that induces early flowering when they are exposed to low temperatures. A major QTL associated with vernalization response was localized on LG3, explaining 55% of the phenotypic variation of this trait [110]. Moreover, Mugabe et al. [109] identified two significant and stable QTLs associated with cold tolerance. A QTL was detected on LG3 at 43.8 cM, and it explained 7.15 to 34.6% of the phenotypic variance, whereas the other QTL was identified on LG8 and explained 11.5 to 48.4% of the variation [109]. In both studies, the same QTL on LG3 was identified, which can be a genomic region of interest associated with cold tolerance and vernalization response in chickpea.

Several efforts have been made to investigate the genetic basis associated with tolerance to salinity in chickpea since it is sensitive to salinity [119] and considering that salt stress is the second major abiotic stress [120]. For example, Vadez et al. [111] identified a QTL for seed yield on LG3, explaining 19% of the variation under salinity conditions. Another QTL for flowering time was located on LG4 and explained 18.5–34.4% of the phenotypic variation. Finally, a cluster of QTLs for seed yield components on LG6 was identified, including a QTL for seed number, which explained 37% of the variation. Subsequently, two important genomic regions on LG5 at 28.6 cM and on LG7 at 19.4 cM containing QTLs for different traits related to yield under salinity were documented, which explained between 12% and 17% of the phenotypic variation [112]. Recently, Soren et al. [121] identified two QTL clusters on LG3 and LG6 containing major QTLs for yield and yield component traits under salinity stress, which explained from 8.8% to 28.4% of the phenotypic variance for this trait. In addition, genomic regions on LG4 were associated with major QTLs for yield under salinity, which explained between 22.6% and 48.5% of the phenotypic variation [113]. These regions on LG4 have also been reported to be associated with drought tolerance in chickpea [105,114]. Therefore, they are of great importance for the breeding programs of chickpea, which would enable the development of highly productive and climate-resilient cultivars by marker-assisted selection.

In practice, the use of genomics tools in breeding programs has allowed the development of new chickpea cultivars through approaches such as marker-assisted backcrossing (MABC), marker-assisted recurrent selection (MARS), and genomic selection (GS), with the MABC approach extensively used to develop improved cultivars of chickpea with higher yield and tolerance to abiotic stresses in recent years [122]. MABC allows one to incorporate desirable major genes/QTLs from a donor parent into an elite cultivar or breeding line (recurrent parent), maintaining almost the entire genetic background of the recurrent parent [37]. In this context, the “QTL-hotspot” region has been introgressed into several chickpea cultivars through the MABC method. For example, the genes/QTLs from the ICC 4958 accession have been successfully introgressed into elite Indian cultivars such as Pusa 372, Pusa 362, DCP 92-3, and JG 11 [114,116], improving drought tolerance and increasing seed yield up to 16% compared to controls under drought stress conditions [116]. In fact, the Pusa Chickpea 10216 (derived from Pusa 372) cultivar was released for commercial cultivation in India [116]. Similarly, the “QTL-hotspot” region from ICC 4958 was introgressed into Kenyan cultivars—Chania Desi II and LDT 068 [115], and Chania Desi 1, ICCV10, ICCV 92318 and Saina K1—increasing the levels of drought tolerance and yield over 2.5 t/ha [123]. In Ethiopia, a high-yielding and drought-tolerant chickpea cultivar named Geletu was developed from the cross between JG 11 × ICC 4958 and released for cultivation. This cultivar had an average yield 15% higher than the control variety Teketay [102]. The development of chickpea cultivars using the MABC approach has recently been discussed in detail by Roorkiwal et al. [122]. Finally, the genomic tools are useful for identifying genomic regions (QTLs) linked to genes associated with grain yield and abiotic tolerance, which can be used in genomic-assisted breeding of chickpea.

#### 4.2.2. Transcriptomics in Chickpea

Genome-wide expression profiling is a useful tool for studying genes differentially expressed in plants under different environmental conditions, allowing the identification of candidate genes and revealing the molecular crosstalk of gene regulatory networks among abiotic stress responses [89]. Thus, transcriptomics approaches have been employed in contrasting chickpea genotypes for tolerance to abiotic stresses to obtain critical information about specific genes and their roles related to drought, extreme temperatures, and salinity tolerance. Despite great advances in genomic resources for dissection of abiotic tolerance in chickpea, as mentioned in Section 4.2.1, the function of various candidate genes and their complex regulatory networks controlling abiotic tolerance is limited. In fact, most of the information available about functional genomics is based on tolerance to drought stress [36].

In chickpea, many efforts have been made to discover genes associated with drought stress responses through transcriptomic studies. Preliminary, Mantri et al. [124] revealed that a total of 477 transcripts were differentially expressed under drought, cold, and salinity stress. Moreover, a total of 20,162 transcripts were differentially expressed under drought and salinity [125]. Subsequently, with RNA-seq technology, many candidate genes that respond differentially to drought stress have been identified [126,127,128,129]. For example, Garg et al. [127] identified a total of 4954 and 5545 genes exclusively regulated in drought and salinity tolerant genotypes, respectively. These genes belong to different pathways, mainly metabolic processes, regulation of transcription, protein modification processes, and signal transduction, among others. More specifically, Badhan et al. [128], using leaf tissue from shoots apical meristem from drought-tolerant (ICC8261) and drought-sensitive (ICC283) chickpea genotypes, identified a total of 1562 genes that were differentially expressed in the drought-tolerant genotype. Genes related to ethylene response, MYB-related protein, xyloglucan endotransglycosylase, alkane hydroxylase MAH-like, BON-1 associated, peroxidase 3, cysteine-rich and transmembrane domain, vignain and mitochondrial uncoupling, were up-regulated and down-regulated in the tolerant and sensitive genotype, respectively. Several transcription factors such as AP2-EREBP, bHLH, bZIP, C3H, MYB, NAC, WRKY, and MADS are involved in drought stress response [129]. A comprehensive *C. arietinum* Gene Expression Atlas (CaGEA) containing a total of 15,947 genes differentially expressed across different developmental stages and organs covering the entire life cycle of chickpea was developed [130]. This allowed for identifying four genes (E3 ubiquitin–protein ligase, LRX 2, kinase interacting (KIP1-like) family, and homocysteine S-methyltransferase) in the “QTL-hotspot” region on GL4, which are up-regulated under drought stress [130].

Several studies have been carried out to analyze differentially expressed genes under salinity conditions [124,125,127,131,132,133]. A total of 3053 differentially expressed genes were identified in response to salt stress, where genes coding for cationic peroxidase, asparticase, NRT1/PTR, phosphatidylinositol phosphate kinase, DREB1E, and ERF were significantly up-regulated in the tolerant J11 genotype [131]. In addition, Kumar et al. [133], using the tolerant J11 and ICCV 10 genotypes, identified a total of 21,698 differentially expressed genes, and a total of 4257 genes were categorized into 64 functional groups, mainly related to integral components of membrane, organelle, and cellular anatomical entity. Moreover, significant up-regulation of transcripts encoding potassium transporter family HAK/KUP proteins, MIP/aquaporin protein family, NADH dehydrogenase, pectinesterase, and PP2C family proteins were reported under salt stress conditions [133]. Considering the heat and cold stresses, the APETALA2/ethylene response factor (AP2/ERF) transcription factor and heat-shock protein 90 (HSP90), contributing to heat stress tolerance in chickpea, were documented [134]. Among the transcripts associated with cold tolerance that are up-regulated, phosphate-induced proteins, beta-glucosidase and beta-galactosidase, and sucrose synthase were reported [124]. Finally, the differential expressions of transcription factor genes such as bHLH-type, NAC, ZFP, bZIP, YABBY, HD-Zip, ERF/AP2, and WRKY play a key role in regulating the response to abiotic stress in chickpea.

Finally, transcriptomic studies have identified thousands of genes and their interaction that participate in the molecular response of chickpea to different abiotic and biotic stresses. In fact, an integrated database of chickpea transcriptomes (CTDB: Chickpea Transcriptome Database) was developed for the identification of candidate genes that are useful in breeding programs of chickpea [135]. These candidate genes are subsequently evaluated and validated through functional genomics studies to determine their function and contribution specific to the plant phenotype. In this sense, Table 4 summarizes some of the candidate genes identified in chickpea that are useful in breeding programs for improving traits related to abiotic tolerance.

#### 4.2.3. Proteomics in Chickpea

Proteomics is the systematic analysis of the proteins expressed by the genome. It not only describes entire proteomes at the cell, organ, and/or tissue level, but it also compares proteomes under different stressful environmental factors [136]. Given that the transcriptomic level does not have an exact or constant correlation with the protein functions and their abundance, proteomics may be altered by posttranscriptional modifications [33]. The modulation of proteome composition in chickpea is an inevitable process to cope with the environmental challenges previously described [137]. To date, most of the studies about abiotic stress in chickpea are related to genomic and transcriptomic approaches, and very little information exists on proteomics.

The nuclear proteome has been studied to better understand the inner cell mechanisms for drought response. Bhushan et al. [138] initiated the proteomic approach in chickpea to identify dehydration-responsive proteins in JG-62, a drought-tolerant cultivar. A total of 134 differentially expressed proteins were identified, which were involved in a variety of cellular functions, such as cell wall modification, signal transduction, metabolism, and cell defense and rescue. Subsequently, Pandey et al. [139] found around 205 protein spots to be differentially regulated under dehydration. The mass spectrometry analysis allowed the identification of 147 differentially expressed proteins involved in functions such as gene transcription and replication, molecular chaperones, cell signaling, and chromatin remodeling. Patel and Hemantaranjan [140] then found that dehydrin-responsive proteins (DRPs) are associated with response to drought stress. A leaf proteome study associated with major abiotic stresses (drought, heat, and salt) was performed by Santisree et al. [141]. A total of 590, 248, and 797 differentially regulated proteins were found for drought, heat, and salt stress, respectively. These proteins were associated with the electron transport chain in photosynthesis, amino acid biosynthesis, ribosome synthesis, and secondary metabolite synthesis, which play key roles in inducing heat and drought tolerance in chickpea [141]. Moreover, proteins related to glutamine synthetase, sucrose and proline biosynthesis, and cytosolic fructose-bisphosphate aldolase were up-regulated in *C. reticulatum* under drought stress [142]. Additionally, multiple stress-related cis-acting elements such as ABRE, MYB, and MYC were found in a proteomic study performed on chickpea for drought stress [143].

Regarding heat stress, Makonya et al. [144] identified an up-regulation in proteins related to protein synthesis, intracellular traffic, defense, and transport in the heat-tolerant genotype (Acc#7). Among the proteins are sucrose-phosphate synthase, sucrose-phosphate phosphatase, HSP70, ribulose bisphosphate carboxylase/oxygenase activase, plastocyanin, and protoporphyrinogen oxidase, which have a role in heat tolerance at the flowering growth stage. Moreover, a total of 482 heat-responsive proteins related to heat shock proteins, such as acetyl-CoA carboxylase, pyrroline-5-carboxylate synthase (P5CS), ribulose-1,5-bisphosphate carboxylase/oxygenase (RuBisCO), phenylalanine ammonia-lyase (PAL) 2, ATP synthase, glycosyltransferase, sucrose synthase, and late embryogenesis abundant (LEA) proteins were associated with heat tolerance in the tolerant JG14 genotype [145]. Under salinity stress, Arefian et al. [146] identified 364 differentially expressed proteins, which were associated with photosynthesis (chlorophyll a-b binding protein, oxygen-evolving enhancer protein, ATP synthase, RuBisCO subunits, carbonic anhydrase, and fructose-bisphosphate aldolase), stress responsiveness (HSP70, 20 kDa chaperonin, LEA-2, and ascorbate peroxidase), and protein synthesis and degradation (zinc metalloprotease FTSH 2 and elongation factor Tu).

Proteomics data help identify the molecular markers associated with candidate genes that encode proteins that play important roles in plant abiotic stress responses, which can be used in breeding programs through proteomics-based marker-assisted selection to improve chickpea cultivars in a more accurately and efficiently way than the conventional breeding strategies. Thus, Table 5 shows some proteins or protein functions involved in conferring tolerance to abiotic stresses that can be targeted in chickpea breeding programs.

#### 4.2.4. Metabolomics in Chickpea

While transcriptomic and proteomic studies are very important steps to reveal the complex biological processes related to abiotic stress tolerance, they are still insufficient to understand the global landscape of cellular response shown by plants under abiotic stress since most biological processes are ultimately mediated by cell metabolites [86]. In this sense, metabolomics is an emerging field of “omics” research that focuses on the high-throughput characterization of small molecule metabolites in biological matrices [147]. From the molecular point of view, metabolomics bridges the gap between genotype and phenotype. Metabolic changes underpin plant development and responses to applied stresses, and the metabolic information reflects biological endpoints more accurately than transcript or protein analysis [148].

Few studies have been conducted evaluating the chickpea metabolome under different abiotic stress conditions. For example, Nisa et al. [149] analyzed two genotypes of chickpea, desi and kabuli, grown under rainfed conditions. Metabolites such as oxalic acid, threonic acid, inositol, maltose, and L-proline show significant differences between the genotypes evaluated; a higher amount of these osmoprotectants were found in the desi genotype under rainfed conditions. Therefore, the inositol phosphate metabolism is involved in plant defense mechanisms against the limited water availability for chickpea production. Moreover, Khan et al. [150] reported increased or decreased levels of different metabolites in drought sensitive (Punjab Noor-2009) and drought tolerant (93,127) cultivars, respectively. The metabolites such as L-proline, L-arginine, L-histidine, L-isoleucine, and tryptophan showed increased levels while choline, phenylalanine, gamma-aminobutyric acid, alanine, phenylalanine, tyrosine, glucosamine, guanine, and aspartic acid showed decreased levels in the tolerant genotype. Similar results were obtained by Khan et al. [151], in which the metabolites L-proline, L-arginine, L-histidine, L-isoleucine, and tryptophan were mostly accumulated in the drought tolerant (93,127) genotype after exposure to drought stress. Under salinity conditions, Dias et al. [152] encountered a decrease in the concentration of arabinose, but a large increase in the tricarboxylic acid (TCA) cycle metabolites isocitrate, cis-aconitate, citrate, fumarate, and malate in the Rupali (salt sensitive) genotype after salinity stress. Finally, the integration of the omics approaches mentioned in the previous sections together with metabolomics can identify the QTLs, genes, proteins, and metabolites involved in the expression of a phenotype of interest. It provides a comprehensive understanding of the molecular networks related to plant response to abiotic stresses and their yields in chickpea.

#### 4.2.5. Transgenomics and Genome Editing in Chickpea

In plants, several genes are activated after being exposed to abiotic stresses, generating an increase in the levels of various osmolytes and proteins that may be responsible for conferring tolerance against these stresses. However, to obtain a significant level of tolerance to abiotic stresses, it is necessary to transfer several potentially useful genes to the same genotype [86]. Transgenomics or transgenic technology is the process of introducing genomic clones from a donor species into a recipient species and then screening the resulting transgenic lines for phenotypes of interest [153]. Therefore, it is a targeted gene-based functional genomics tool that offers valuable information to understand the regulatory mechanisms underlying abiotic stress tolerance in plants [154]. In this context, some efficient transformation protocols have been developed and applied in legume crops, including chickpea [155,156,157].

Osmoregulation is one of the main mechanisms conferring abiotic stress tolerance, especially if osmoregulatory genes could be triggered in response to drought, salinity, and high temperatures [52]. Some transgenomics studies have been performed to improve abiotic tolerance in chickpea. For example, Bhatnagar-Mathur et al. [156] used transgenic technology for the introduction of an osmoregulatory gene P5CSF129A under the CaMV35S promoter, which improved drought stress tolerance in chickpea by enhancing proline accumulation. Similarly, the expression of AtDREB1A under the drought inducible Rd29A promoter influenced the mechanisms underlying water uptake, stomatal response, transpiration efficiency, and rooting architecture and enhanced drought tolerance in transgenic chickpea lines [157,158]. On the other hand, the silencing of an HD-Zip I gene, CaHDZ12, resulted in increased sensitivity to salt and drought stresses in chickpea [159].

Plant transformation has provided fundamental insights into the molecular biology of plants. Unfortunately, the transformation and regeneration remain limited for most crops even after more than three decades of technological advances [160]. Genome editing is a revolutionary technology in molecular biology in which a specific target DNA sequence of the genome is altered by adding, removing, or replacing DNA bases. Artificially engineered hybrid enzymes, zinc-finger nucleases (ZFNs), transcription activator-like effector nucleases (TALENs), and the CRISPR (clustered regularly interspaced short palindromic repeats)–Cas (CRISPR-associated protein) system are being used for genome editing in plants [161]. To date, the only study using genomic editing in chickpea was carried out by Badhan et al. [162], where two genes associated with drought tolerance, 4-coumerate ligase (4CL) and Reveille 7 (RVE7), were successfully edited (knockout) by CRISPR/Cas9 to increase drought stress tolerance in chickpea. Therefore, the knockout of these genes using genome editing is a novel approach that can be used in the development of drought-tolerant cultivars of chickpea in the future.

## 5. Future Perspectives and Conclusions

The shifts in rainfall patterns and terminal heat stress are likely the factors most affecting the yield and quality of cultivated chickpea. In this context, one of the main challenges faced by crop breeders is to generate high-yielding cultivars that can cope with climate change scenarios. In this sense, pre-breeding plays a key role in the genetic improvement of chickpea due to the narrow genetic base of the cultivated species. The introgression of desirable genes/alleles from wild germplasm into cultivated chickpea can improve the tolerance levels to abiotic stress and their yields, which is needed to maintain food security for the upcoming years. Therefore, great efforts have been made to develop chickpea cultivars that are tolerant to different abiotic stresses, which have been achieved through the combination of conventional and molecular (omics) breeding methods. However, there are still efforts to be made.

In the future, the selection of new cultivars should be based on a complete phenotypic and genetic description of the materials to be used as parental material in breeding programs. This can be based on new high-throughput phenotyping techniques [163], such as the characterization of plant canopy temperature and root system architecture in heat and drought stress experiments [164]. The whole genetic characterization of parent material should be one of the cornerstones in the future. DNA sequencing methods should be faster, more accurate, and cheaper to obtain the genetic information of the plants to be used in breeding programs. Additionally, due to the increased utilization of genomics and environmental data, the use of enviromics approaches, which involve the application of envirotyping methods to describe the performance of a plant along different gradients of many environmental variables that the plant is exposed to during its growth cycle [165], is highly useful in modern breeding programs considering that the environmental effects of global warming are variable in different geographical areas. In addition, efforts to improve the concentration of nutrients in grains, especially micronutrients such as Fe and Zn, must also be considered and enhanced. A powerful tool for this purpose is the selection of seeds using ionomics, for example, with the use of micro-X-ray fluorescence spectroscopy, which allows the determination and mapping of the elemental nutritional concentrations and their distributions within the grains [166,167]. Simultaneously, data mining should be faster and end-user friendly, so that plant breeders can directly analyze and interpret the information to decide during the pre-selection process which plant material should be used in the subsequent breeding stages. The use of artificial intelligence to link the information obtained at different scales is also advancing and should be one of the objectives in the present decade [168]. Moreover, the preservation of plant genetic resources should be a priority since it contains the genetic variability to cope with future climatic adversities.

Finally, the integration of the genetic improvement approaches reviewed in this work, together with the high-throughput phenotypic evaluation, will permit a better understanding of the molecular, biochemical, and physiological mechanisms involved in the plant response to stressful environmental conditions. This allows a more precise and effective selection/introgression of genotypes and/or genes/alleles that contribute to increased grain yields considering the effects of climate change in the medium and long term on chickpea production.

## Figures and Tables

**Table 1:** Wild Cicer species as sources of alleles to abiotic stress tolerance in chickpea.

Wild Species	Source of Alleles to Abiotic Stress Tolerance	References
*C. reticulatum*	Drought, heat, cold, and salinity	[71,72,73,74,75]
*C. echinospermum*	Cold	[72,73,75]
*C. bijugum*	Cold	[73,75]
*C. judaicum*	Cold and drought	[75,76]
*C. pinnatifidum*	Drought and heat	[71]

**Table 2:** Mutant cultivars of chickpea approved by the Joint FAO/IAEA Division of Nuclear Techniques in Food and Agriculture.

Cultivar Name	Country	Registration	Improved Traits
Hyprosola	Bangladesh	1981	Early maturity, more pods, higher harvest index, higher planting density, higher yield.
CM 72	Pakistan	1983	Resistance to chickpea blight (*Ascochyta rabiei*) and high yield
Kiran	India	1984	Erect plant type, increased pod number, high yield, early maturity, and salt tolerance
Pusa 417 (Girnar)	India	1985	Wilt and pod borer resistant
Pusa 413 (Atul)	India	1985	High yield, high pod number, early maturity, wilt resistance, and moderate resistance to biotic stress
Pusa 408 (Ajay)	India	1985	High yield, blight resistance, semi-erect, early maturity, and improved plant architecture
NIFA-88 (CM-1918)	Pakistan	1990	Moderate resistance to *Ascochyta rabiei*, earlier maturity, high yield, higher nitrogen amount fixation
Line 3	Egypt	1992	High yield
CM-88	Pakistan	1994	Resistance to ascochyta, resistance to Fusarium, and high yield
NIFA-95	Pakistan	1995	Resistance to bacterial blight
Binasola-2	Bangladesh	1998	No information
CM-98	Pakistan	1998	Resistance to Ascochyta blight and Fusarium wilt
CM 2000	Pakistan	2000	High yield and resistance to diseases
Hassan-2K	Pakistan	2000	High yield, higher protein content, and resistance to blight and wilt
Binasola-4	Bangladesh	2001	Higher seed yield, medium seed size, and bright seed coat color
Binasola-3	Bangladesh	2001	Early maturity, erect plant type, larger seed size, and rough seed coat
BGM 547	India	2005	High yield, bold grain size, and moderate resistance to abiotic factors
THAL-2006	Pakistan	2006	Tolerance to blight, tolerance to moisture stress, and bold seed size
TAEK-SAGEL	Turkey	2006	High yielding and Ascochyta resistance
Pusa 547	India	2006	High yield, good cooking quality, tolerance to Fusarium wilt, stunt virus, and root rot
CM-2008	Pakistan	2008	Improved seed size, resistance to wilt, and high yield
Binasola-6	Bangladesh	2009	Size and color of seed, and higher seed yield
Binasola-5	Bangladesh	2009	Size and color of seed, and higher seed yield
Binasola-7	Bangladesh	2013	Higher seed yield, medium seed size, deep green leaves, and brown seed coat color
Binasola-8	Bangladesh	2013	Higher seed yield, medium seed size, and attractive straw seed coat color
Binasola-10	Bangladesh	2016	Straw seed coat color, bolder seed size, and higher seed yield
Binasola-9	Bangladesh	2016	Cream seed coat color (kabuli type), bolder seed size, and higher seed yield

**Table 3:** List of some major QTLs associated with traits related to abiotic tolerance in chickpea.

Stress	Trait	Linkage Group (LG)	Markers/Locus	R^2^	Cross/ Genotypes	Reference
Drought	PH	LG1	H5A08-TA8	24	ILC 588 × ILC 3279	[61]
	DTS	LG3	TA6-NCPGR12	27		
	DTF	LG3	TA6-NCPGR12	22		
	DTM	LG3	TA6-NCPGR12	33		
	HI	LG3	TA6-NCPGR12	25		
	DTS	LG3	H6C-07	23.3	ILC 588 × ILC 3279	[101]
	PN	LG3	H6C-07	22.7		
	DTF	LG3	H6C-07	24.2		
	DTM	LG3	H6C-07	20.3		
	RW	GL4	ICCM0249	58.2	ICC 4958 × JG 11	[102]
		GL4	STMS11	58.2		
	PH	GL4	NCPGR127–CPGR21	30.2	ICC 4958	[103]
	DTF	GL4	NCPGR127–TAA170	24.49		
	100SW	GL4	NCPGR127–NCPGR21	58.2		
	PPP	GL4	NCPGR127–NCPGR21	23.18		
	SPP	GL4	TAA170–NCPGR21	42.07		
	DTF	GL8	NCPGR164–CaM1918	26.87		
	PH	GL4	Ca4_12982420–TAA170	10.78–26.91	ICC 4958 × ICC 1882	[104]
	100SW	LG4	Ca4_13687456–TAA	10.12–60.41		
	DTM	LG7	NCPGR164–Ca8_3050452	10.11–47.43		
	PB	GL8	CaM0812–NCPGR164	10.05–34.57		
	HI	LG8	NCPGR164–Ca8_3050452	10.14–25.94		
	PPP	LG8	Ca4_13687456–TAA17	10.73–32.34		
	R/PDW	LG4	bin_4_13393647–bin_4_13547009	20.09	ICC 4958 × ICC 1882	[105]
	SDW	LG4	bin_4_13393647–bin_4_13547009	25.22		
	PH	LG4	bin_4_13239546–bin_4_13378761	41.76		
	100SW	LG4	bin_4_13239546–bin_4_13378761	59.83		
	DTM	LG7	bin_7_12870961–bin_7_12856579	45.38		
	DTF	LG8	bin_8_6034209–bin_8_5984553	44.76		
	PH	LG4	Bin_4_13239546-Bin_4_13378761	36–39	ICC 4958 × ICC 1882	[63]
	PH	LG4	Bin_4_13239546-Bin_4_13378761	23		
	PV	LG4	Bin_4_13239546-Bin_4_13378761	53		
Heat	GY	LG5	Ca5_44667768-Ca5_46955940	16.56	ICC 4567 × ICC 15614	[106]
	CC	LG6	CPGR206-H3G031	17.4	DCP 92-3 × ICCV 92944	[107]
	MSI	LG5	NCPGR267	16.5	71 desi genotypes	[108]
	MSI	LG6	H2L102	15.5		
	MSI	LG7	TS 53	22.2		
Cold	SS	LG1	999_1	15.93	ICC 4958 × PI 489777	[109]
	CT	LG3	2574 _ 3	24–34.7		
	SS	LG4	3594_4	29.41		
	PH	LG4	474 0 _ 4	20.21		
	CT	LG8	9604_8	32.37–48.41		
	PH	LG8	9648_8	19.97		
	VER	LG3	H1F14-TA64	47.9–54.9	ICC 4958 × PI 489777	[110]
Salt	SDW	LG5	TS46–NO_X_1	26.6	ICCV 2 × JG 62	[111]
	SDW	LG6	TA186–TA46	23.3		
	SDW	LG6	TR20s–TA46	21.4		
	SN	LG6	TR20s–TA46	25.1		
	100SW	LG6	GA137–GA25	43.2		
	100SW	LG7	TA11–TA42	27.6		
	DTM	LG1	CaM1301-CKAM1971	66.75	ICCV 2 × JG 11	[112]
	DTF	LG4	CKAM0003-CKAM1003	22.6		
	DTM	LG4	CKAM0003-CKAM1003	59.95		
	HI	LG4	CKAM0003-CKAM1003	49.13		
	100SW	LG5	CaM0038-CaM0463	17.42		
	DTF	LG5	CaM0463-ICCM272	24.98		
	DTM	LG5	CaM0463-ICCM272	40.69		
	100SW	LG5	CaM0463-ICCM272	33.4		
	HI	LG5	CaM0463-ICCM272	29.85		
	GY	LG7	CaM2031-CKAM0165	16.99		
	PN	LG7	CaM2031-CKAM0165	24.86		
	SN	LG7	CaM2031-CKAM0165	16.86		
	DTF	LG8	CKAM1903-CKAM0343	37.75		
	DTM	LG8	CKAM1903-CKAM0343	56.87		
	HI	LG8	CKAM1903-CKAM0343	47.23		
	BM	LG1	SNP27-SNP23	16.5	Rupali × Genesis 836	[113]
	WUE	LG1	DArT85-DarT78	46.3		
	BM	LG4	SNP14_C14_12_74-SNP15_C14_13_06	48.5		
	PN	LG4	SNP201-SNP2_Ca4_75	21.3		
	PN	LG4	DarT417-SNP203	15.1		
	SN	LG4	SNP201-SNP2_ Ca4_75	28.5		
	SN	LG4	DarT417-SNP203	23.2		
	100SW	LG4	SNP14_C14_12_74-SNP15_C14_13_06	22.6		
	100SW	LG4	SNP14_C14_12_74-SNP15_C14_13_06	34.4		
	GY	LG4	SNP201-SNP2_ Ca4_75	22		
	GY	LG5	DarT595-DarT553	17.9		
	100SW	LG5	DarT595-DarT553	21.8		

PH: plant height, DTS: drought tolerance score, DTF: days to flowering, DTM: days to maturity, HI: harvest index, PN: pods number, SN: seed number, RW; root weight, PPP: pods per plant, SPP: seeds per pod, PW: primary branches, R/PDW: root/plant dry weight, PV: plant vigor, CC: chlorophyll content, 100SW: 100-seed weight, GY: grain yield, SS: seed size, PH: plant height, BM: biomass, CT: cold tolerance, VER: vernalization, MSI: membrane stability index, SDW: shoot dry weight, WUE: water use efficiency. R^2^: percentage of explained phenotypic variance.

**Table 4:** Some functional genomics studies carried out in chickpea for traits related to abiotic tolerance.

Category	Genotype Tolerant	DEG	Candidate Genes	Reference
Drought	ICC8261	1562	Ethylene response, MYB-related protein, xyloglucan endotransglycosylase, alkane hydroxylase MAH-like, BON-1 associated, peroxidase 3.	[128]
	BG-362 and P-256	1624	AP2-EREBP, bHLH, bZIP, C3H, MYB, NAC, WRKY, and MADS	[129]
	ICC 4958	15,947	E3 ubiquitin-protein ligase, LRX 2, kinase interacting (KIP1-like) family, and homocysteine S-methyltransferase	[130]
Salinity	J11	3053	Cationic peroxidase, asparticase, NRT1/PTR, phosphatidylinositol phosphate kinase, DREB1E and ERF	[131]
	J11 and ICCV 10	21,698	HAK/KUP proteins, MIP/aquaporin protein family, NADH dehydrogenase, pectinesterase, and PP2C family proteins	[133]
Heat	ICCV 92944, ICC 1356 and ICC 15614	147	AP2/ERF and HSP90	[134]
Cold	Sonali and ILC 01276	57	Phosphate-induced proteins, beta-glucosidase and beta-galactosidase, and sucrose synthase	[124]

DEG: differentially expressed genes.

**Table 5:** Proteins or protein functions involved in conferring tolerance to abiotic stresses to chickpea.

Category	Genotype Tolerant	DEP	Proteins or Protein Function	Reference
Drought	JG-62	134	Cell wall modification, signal transduction, metabolism, and cell defense and rescue.	[138]
	JG-62	147	Gene transcription and replication, molecular chaperones, cell signaling, and chromatin remodeling.	[139]
	ILC482	24	Glutamine synthetase, sucrose and proline biosynthesis, and cytosolic fructose-bisphosphate aldolase.	[142]
Heat	Acc#7		Sucrose-phosphate synthase, sucrose-phosphate phosphatase, HSP70, ribulose bisphosphate carboxylase/oxygenase activase, plastocyanin, and protoporphyrinogen oxidase.	[144]
	JG14	482	Acetyl-CoA carboxylase, P5CS, RuBisCO, PAL 2, ATP synthase, glycosyltransferase, sucrose synthase, and LEA proteins.	[145]
Salinity	Flip 97-43c	364	Chlorophyll a-b binding protein, oxygen-evolving enhancer protein, ATP synthase, RuBisCO subunits, carbonic anhydrase, fructose-bisphosphate aldolase, HSP70, 20 kDa chaperonin, LEA-2, ascorbate peroxidase, zinc metalloprotease FTSH 2, and elongation factor Tu.	[146]

DEP: differentially expressed proteins.
